# Automated Detection and Scoring of Tumor-Infiltrating Lymphocytes in Breast Cancer Histopathology Slides

**DOI:** 10.3390/cancers15143635

**Published:** 2023-07-15

**Authors:** Mohammad Yosofvand, Sonia Y. Khan, Rabin Dhakal, Ali Nejat, Naima Moustaid-Moussa, Rakhshanda Layeequr Rahman, Hanna Moussa

**Affiliations:** 1 Department of Mechanical Engineering, Texas Tech University, Lubbock, TX 79409, USA; mohammad.yosofvand@ttu.edu (M.Y.); rabin.dhakal@ttu.edu (R.D.); 2 Breast Center of Excellence, Department of Surgery, Texas Tech University Health Sciences Center, Lubbock, TX 79430, USA; sonia.khan@ttuhsc.edu (S.Y.K.); rakhshanda.rahman@ttuhsc.edu (R.L.R.); 3 Department of Civil, Environmental, & Construction Engineering, Texas Tech University, Lubbock, TX 79409, USA; ali.nejat@ttu.edu; 4 Department of Nutritional Sciences, Texas Tech University, Lubbock, TX 79409, USA; naima.moustaid-moussa@ttu.edu; 5 Obesity Research Institute, Texas Tech University, Lubbock, TX 79409, USA

**Keywords:** tumor-infiltrating lymphocytes, immunotherapy, deep learning modeling, whole cancer slide imaging, digital pathology

## Abstract

**Simple Summary:**

Tumor-infiltrating lymphocytes have gained a critical role in the newly developed cancer immunotherapy methods. Traditionally, pathologists and researchers count and score these lymphocytes to evaluate the patient’s response to the treatments. However, scoring the lymphocytes is conducted microscopically and highly depends on the pathologist and researcher’s experiences. In this manuscript, we have proposed and developed an automated method, using a two-stage deep learning model to score the lymphocytes in breast cancer histopathology slides. We further verified the accuracy of our method by statistically comparing results from expert pathologists to the output from our developed model.

**Abstract:**

Detection of tumor-infiltrating lymphocytes (TILs) in cancer images has gained significant importance as these lymphocytes can be used as a biomarker in cancer detection and treatment procedures. Our goal was to develop and apply a TILs detection tool that utilizes deep learning models, following two sequential steps. First, based on the guidelines from the International Immuno-Oncology Biomarker Working Group (IIOBWG) on Breast Cancer, we labeled 63 large pathology imaging slides and annotated the TILs in the stroma area to create the dataset required for model development. In the second step, various machine learning models were employed and trained to detect the stroma where U-Net deep learning structure was able to achieve 98% accuracy. After detecting the stroma area, a Mask R-CNN model was employed for the TILs detection task. The R-CNN model detected the TILs in various images and was used as the backbone analysis network for the GUI development of the TILs detection tool. This is the first study to combine two deep learning models for TILs detection at the cellular level in breast tumor histopathology slides. Our novel approach can be applied to scoring TILs in large cancer slides. Statistical analysis showed that the output of the implemented approach had 95% concordance with the scores assigned by the pathologists, with a *p*-value of 0.045 (n = 63). This demonstrated that the results from the developed software were statistically meaningful and highly accurate. The implemented approach in analyzing whole tumor histology slides and the newly developed TILs detection tool can be used for research purposes in biomedical and pathology applications and it can provide researchers and clinicians with the TIL score for various input images. Future research using additional breast cancer slides from various sources for further training and validation of the developed models is necessary for more inclusive, rigorous, and robust clinical applications.

## 1. Introduction

Breast cancer is a common disease among women. In the United States, 12 percent of women are diagnosed with invasive breast cancer, which is the cause of second-highest number of cancer cases and the highest death rate after lung cancer. The rate of breast cancer diagnoses has increased over the past few decades [1] and has been linked to both genetic and environmental factors [2]. Thus, rapid advances in breast cancer diagnosis and treatment are urgently needed to improve health and the quality of life of a large segment of the population.

Several methods are used for breast cancer treatments including surgery, hormonal therapy, and radiation therapy [3]. One of the newly developed methods for breast cancer treatment is immunotherapy, where immune cells are extracted from the patient’s body and grown in the laboratory, then injected back into the patient to detect and kill tumor cells [4]. These immune cells are called stromal tumor-infiltrating lymphocytes (sTILs). The higher the number of sTILs in a patient’s body, the more effective the immunotherapy is [5]. Therefore, quantifying and calculating the number of sTILs in cancer samples from patients is a very important part of determining the clinical treatment.

Quantification and detection of sTILs in hematoxylin and eosin (H&E)-stained histology images are nowadays considered a predictive and prognostic tool in early stage HER2-positive (HER2+) and triple-negative breast cancer [6]. Stromal TILs are host immune cells located inside the boundary of a tumor but not in direct contact with them [7]. The percentage of sTILs is a key factor in treatment; a higher sTILs percentage predicts a better response by the patient’s body to immunotherapy cancer treatment. This percentage is calculated by dividing the area of sTILs cells over the total area of the stroma of the tumor [8]. The procedure used to calculate this percentage is described as immunoediting theory with clinical data supporting it [9]; thus gaining more attention in breast cancer research [10,11].

International Immuno-Oncology Biomarker Working Group (IIOBWG) on Breast Cancer has made recommendations for assessing sTILs in breast cancer tumors and the International sTILs Working Group 2014 endorsed the guidelines for evaluation of stromal tumor-infiltrating lymphocytes in breast tumors [12]. This was followed by an update of the recommendations for assessing sTILs by the same committee in 2018 which included morphological evaluation of sTILs in breast tumors [13].

Morphological evaluation guidelines recommended by the International Immuno-Oncology Biomarker Working Group on Breast Cancer are a significant tool for researchers to assess sTILs in breast cancer. These guidelines include recommendations on the color, shape, and size of sTILs to calculate the percentage sTILs within the borders of the residual tumor bed [14]. Pathologists follow these guidelines and look at the slides and designate a sTILs percentage. They look at different sections of the slides and give each section a score, which they average and assign as the sTILs score. Despite these efforts made to regulate the TILs scoring procedures, estimating the sTILs percentage in a breast tumor slide can vary among researchers and pathologists, in particular, depending on experience. For a given slide, pathologist A might estimate the sTILs percentage differently from pathologist B, due to their non-homogeneous experience with the guidelines or assessment of histology slides. In a recent study, conducted by Kos et al., sixty pathologists were tasked to score sTILs in some breast tumor samples [15]; the results showed that for one slide, the scores given by pathologists differed by 10%, which is a high level of variability.

Usage of computer methods, such as artificial intelligence (AI) and machine learning (ML) has gained significant traction in medical applications, such as analyzing magnetic resonance imaging (MRI), CT scans, and drug discovery due to their accuracy and pace. Breast cancer treatments have also been improved by using various diagnostic machine learning treatments [16,17]. Machine learning based methods can be used for a wide range of applications in cancer treatments encompassing predictive models for survival predictions in breast cancer [18,19] and breast cancer recurrence based on the existing data from previous patients [20]. Artificial intelligence and computer methods also can be used for diagnosis and prognosis purposes in breast cancer research together with cancer treatment methods [21,22]. Bejnordi et al. [23] developed a deep learning algorithm in a research challenge competition in the Netherlands in 2016 to assess lymph node metastases in slides from women diagnosed with breast cancer. The best algorithm developed in the competition could outperform pathologists in a simulation exercise which shows the importance of machine learning methods in medical diagnosis. In another application of machine learning methods in breast cancer research, Tellez et al. [24] used a convolutional neural network (CNN) algorithm to detect mitotic tumor cells in H&E slides of breast cancer. Further, Amgad et al. [25] segmented breast cancer images using fully convolutional networks with high accuracy; such networks were also used to predict cancer diagnosis results from histologic images and genomics data by Mobadersany et al. [26].

Considering the importance of scoring the stroma TILs, we implemented CNN-based deep learning models on breast cancer slides provided by the Breast Center of Excellence at the Texas Tech University Medical Center. Through training sessions with the pathologists, and following the guidelines from (IOBWG), the stroma and TILs in the breast tumor slides were identified and annotated. In this manuscript, we used 63 large-size tumor slides available for research purposes to implement deep learning models for scoring TILs.

Here, we will first introduce the dataset used to develop the toolkit software for sTILs detection and scoring in the cancer slides. Then, the deep learning model is discussed, and challenges are highlighted. Next, results from the training of the models used to segment cancer slides and detect TILs are presented. This will be followed by model validation to facilitate its future use by practitioners. We will conclude the paper by highlighting study limitations and the future work needed to further translate this work into practice.

## 2. Dataset Description

The Breast Cancer Center of Excellent at Texas Tech University Medical Research Center (TTUMRC) granted access to 63 slides of breast tumors. All histology slides used in this project were anonymously coded, and subjects were recruited under the protocol number “L 21-175”, approved by the Texas Tech Health Sciences Center Institutional Review Board (IRB) as an exempt protocol. The current work is a retrospective analysis from archival tissue and clinical information with no identifying information was used. 

**Figure 1:**
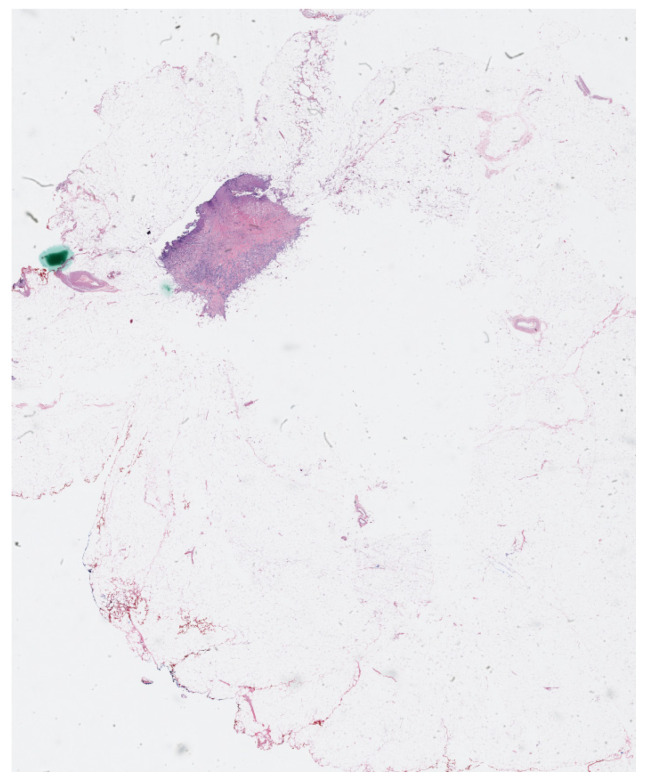
Digital tumor histology slide image (55,240 × 78,104 pixels) at zoom level 1×.

The physical imaging slides that contained biopsy cancer samples were scanned and digitalized to create whole histopathology slides at high resolutions using OptraSCAN services (OptraSCAN Inc., San Jose, CA, USA). The dataset includes 63 whole slide imaging files in “JP2” format which is a compression method that can save biomedical images with various zoom levels. The digital biomedical slides used in the manuscript have different zoom levels ranging from 1×, which is the regular zoom level, up to 40× zoom level. A sample of the digitalized cancer slides is shown in Figure 1.

Due to the very large size of the whole cancer imaging slides of the dataset, each slide was divided and cropped into smaller images; each smaller image is called a tile or patch and these tiles are fed to the convolution neural networks. The patch images were used to create two datasets: the tissue dataset, and the TILs dataset.

### 2.1. Tissue Dataset

In addition to the size issue of the .jp2 dataset, not all parts of the cancer imaging slides should be included in TILs scoring. It is important to accurately detect the stroma area as recommended by the IIOBWG on Breast Cancer to score the TILs; therefore, we created a dataset of tissue images that are used in segmentation algorithms to segment the whole images fed into the CNN models to determine the stroma area for the slides.

In this dataset, we have two classes to segment: the stroma class and the background. The stroma areas are identified at the invasive area of cancer and selected randomly to create a diverse dataset from all 63 cancer slides, then the stroma area of the slides is annotated manually to create the masks required for segmentation by CNNs. The tissue dataset is made of image patches. The tissue dataset of the area of interest includes 1600 images with the corresponding masks to determine the stroma tissue in the images. The images in the dataset are in RGB color space and each channel (red, green, and blue) has a value between 0 and 255. Each mask has the exact size as the images, and it is a grayscale image. In masks, the stroma area of the images is marked with white color (value = 255). The rest of the image which includes the background and other areas, such as cancer tissue that are not considered in TILs scoring by IIOBWG recommendations is assigned a value of 0.

### 2.2. TILs Dataset

After locating the stroma tissue area in the images, we used image patches to annotate the TILs. To annotate the TILs for the second data set, IIOBWG recommendations were employed to determine whether a cell is a TIL or not. Also, the determination process was conducted with the help of pathologists from the Breast Center of Excellence at TTUMRC to ensure the accurate creation of the dataset. 

To annotate the TILs, the coco format was used. We used an online automatic annotation creator to annotate all the TILs in the stroma areas and create the JSON file with the coco annotation format automatically; patch images with a 40× zoom level were used to create the TILs annotation dataset, each annotation consists of a 16 × 16 bounding box around the TILs and the annotation id as well as the annotation location were automatically stored in coco format. We created a dataset of about 12,000 TILs annotated for the 1600 images that were used for the tissue segmentation part. For this dataset, the cells are placed at the center of the squares around the objects. In the TILs dataset, we only have one category id, which indicated that the identified cells belong to the TILs category; per recommendations from the IIOBWG, other cells, such as cancer cells, eosinophils, neutrophils, mitosis, etc., were not annotated.

## 3. Proposed Models

After creating the dataset, various CNN-based architectures were employed to segment the images to find the stroma area and detect the TILs. First, a simple 2 layers CNN was implemented to test the dataset. The input images for the model were resized to 128 × 128 × 3, and we set the kernel for the CNN to be a 3 × 3 matrix. The CNN-based architectures have the capability to extract the most important features in an image and are shown to be efficient in pathology and cancer image analysis and segmentation. The simple CNN model that was used as a test process, provided acceptable results for the segmentation dataset. When used consecutively, U-Net can be implemented as a pre-processing step to segment the image and extract the region of interest (ROI), the stroma area. The resulting ROI was then passed to the Mask R-CNN model for further object detection and classification of the TILs. This combined approach of two consecutive models can increase the precise level of localization and identification of the TILs, leveraging the strengths of both the models.

Furthermore, using U-Net as the initial segmentation method followed by R-CNN model as the object detection tool for the segmented region of interest enhances the overall performance and accuracy of the model. The consecutive usage of U-Net and R-CNN is more accurate when precise object localization and identification are critical for the deep learning model, such as detecting tumor cells in pathology. Dogan et al. used a two-stage model including Mask R-CNN and U-Net to automatically segment pancreas in medical images [27]. Consecutive combination of U-Net and R-CNN was used to segment the whole heart and it was shown that combining the two models can improve the accuracy of and overall performance of the model [28,29]. Konopczyński et al. used a Mask R-CNN and U-Net to detect cells in cancer slides and showed the performance of the two models are enhanced when a combined model is used [30]. Therefore, to increase the overall accuracy of the deep learning model as well as following the established procedure of scoring TILs by pathologists, a combined two-stage model was selected for this research. First, a U-Net network was used to segment the stroma area in the images, and then a Mask R-CNN model was trained to detect the TILs in the images.

### 3.1. U-Net Architecture

A U-Net architecture network was implemented to segment the tissue images with the corresponding masks. U-Net is a deep CNN model that was developed by Ronneberger et al. specifically for biomedical segmentation tasks [31]. U-Net is an encoder–decoder model that has two stages. The first stage of the U-Net is called the contracting path, in which we decreased the size of the image, while significantly increasing the number of layers. In the next stage, we increased the size of the image, until it reached its original size, while decreasing the number of layers; this stage is called expanding path. At the end of the architecture, we applied an identity matrix to the image to create an output with the exact size of the original image to ensure that the objects and cells in the biomedical images are at their original size. This feature of the U-Net is very desirable in biomedical and pathology image segmentation tasks, which makes the U-Net a popular choice [32]. Therefore, we used a U-Net to train the model for the stroma detection task.

**Figure 2:**
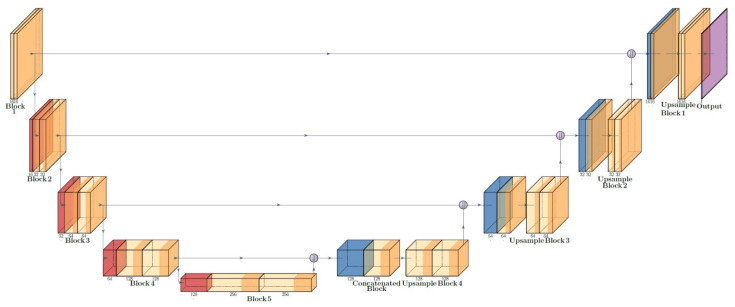
Architecture of the constructed U-Net.

The input images are resized to 128 × 128 × 3 pixels in RGB color space before they are fed to the U-Net. Both tissue images and mask images are normalized by dividing their pixel values by 255 and the input matrices have a value between 0 and 1. The first block of the U-Net increases the image to 16 layers. The contracting path contains 5 blocks and at each block kernel layers are doubled; at the deepest layers, there are 256 kernel layers, and it has an 8 × 8 size. In the expanding path, the size of the feature maps increases to 128 × 128, and a 1 × 1 kernel is applied to the output to create the 128 × 128 × 1 desired output that can be compared with the masks. The U-Net architecture is shown in Figure 2.

### 3.2. Mask R-CNN Model

To perform the object detection task efficiently, the Mask R-CNN libraries in python were employed [33]. In the Mask R-CNN model, not only boxes around objects are determined, but also a mask for each object is created that contains various information about the detected object, including the area of the object. This feature allows the Mask R-CNN model to detect and calculate the area of the TILs in this research in a very aligned method with the IIOBWG guidelines; thus, it was selected for the TILs detection tasks.

**Figure 3:**
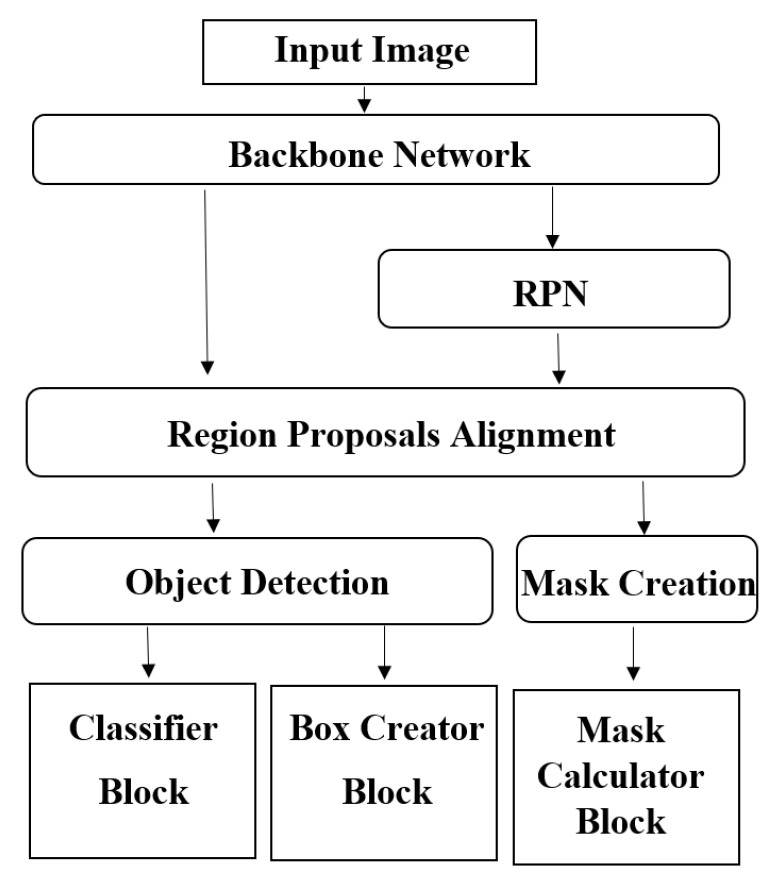
The architecture of Mask R-CNN and the corresponding blocks.

The annotations created for the TILs in coco format were implemented as masks to perform the detection using the Mask R-CNN model. A Resnet 101 network was used as the backbone architecture of the Mask R-CNN. Resnet 101 is a CNN model which is 101 layers deep and contains 138 million learnable parameters [34]. Resnet 101 can be implemented and trained very efficiently, which makes it a proper selection for the training of the Mask R-CNN in the paper. We also imported the pre-trained weights from the “mask_rcnn_coco.h5” to speed up the learning process. The Mask R-CNN model used in the TILs detection task is presented in Figure 3.

## 4. Results and Discussion

To develop the TILs detection toolkit software, the U-Net was trained to segment the stroma areas. The performance of the suggested U-Net model was investigated for the segmentation of stroma from the background for different hyperparameters to achieve the highest rate possible; this step plays a critical role in the model development as it will be used for finding the appropriate area of the cancer slides that should be used in the scoring process. Due to the small number of available slides, we trained the whole dataset for the segmentation purpose to get the highest possible accuracy. Then, the performance of the Mask R-CNN model in detecting the TILs in the stroma area was evaluated and tested on the dataset. Finally, we developed graphical user interface (GUI) to provide an automatic pipeline for scoring TILs in cancer images for the researchers in the Breast Center of Excellence at TTUHSC.

### 4.1. Stroma Segmentation

The images and masks for stroma segmentation were fed to the U-Net architecture described in Section 3.1. We trained the U-Net model with different hyperparameters and loss functions to achieve the best results; in the training procedure, first, we shuffled the training images from all 63 imaging slides to diversify the dataset, then 20% of the images were randomly selected to make the validation dataset for each training. This step was used to tune the U-Net model and then the final training process was conducted with the whole dataset. The training process was conducted in Python version 3.9.12; the TensorFlow library version and Keras library version were both 2.10.0. An NVIDIA GeForce RTX 3070 was employed to run the training as it increases the speed of the training process. A comparison between CPU and GPU processing time for one iteration is shown in Table 1.

**Figure 4:**
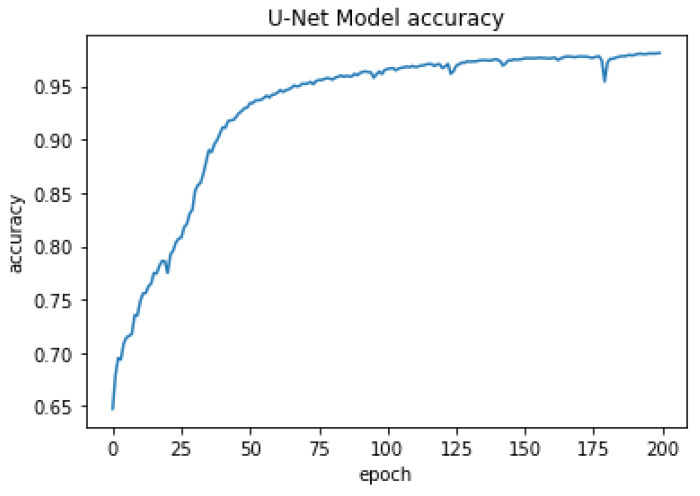
Model accuracy in stroma segmentation.

**Figure 5:**
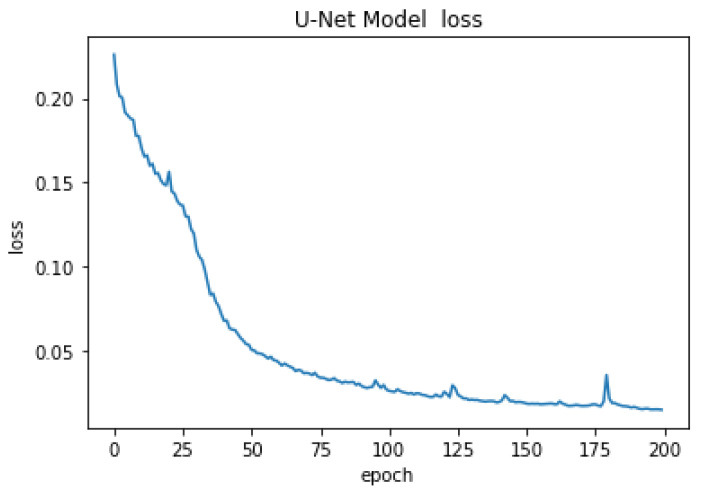
Loss values in stroma segmentation for the U-Net model.

After training the model for the final step, the U-net architecture could reach an accuracy of 80% after 25 epochs, 90% after 40 epochs, and the final accuracy was 98.09% after 200 epochs. The optimizer uses a lower decay rate (0.9) for the beginning steps of the training process, as a result, the model can converge to the results quickly, and then it uses the higher decay rate (0.99) which helps the model to converge to the final solution with more accuracy without getting overfitted. The accuracy of the U-Net model is presented in Figure 4 for 200 epochs. The high accuracy of the model helps us segment the stroma tissue with precision which can be used for TILs detection. The loss values for the model are presented in Figure 5 for the whole 200 epochs. The mean square error loss function used to calculate the loss values is calculated as:

(1) $$L=\frac{1}{n}\sum_{i}^{n}{\left(y_{i}-y_{t,i}\right)}^{2}$$

where, the *y_i_* is the U-Net output and the *y*_*t*,*i*_ is the target.

**Figure 6:**
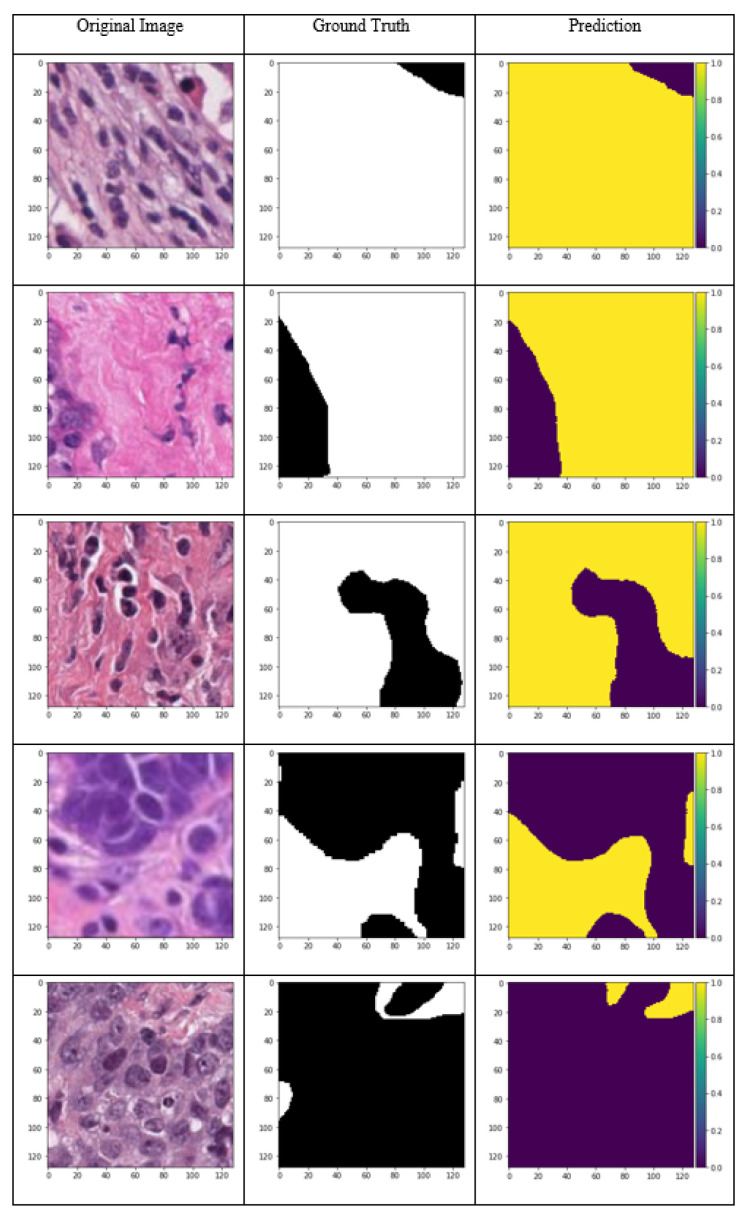
Model results for different sample images with the ground truth (Original magnification: 40×).

The model predictions for the input samples are presented in Figure 6. Different samples from high stroma percentage to high background samples, including cancer areas are shown to investigate the performance of the U-Net model for the dataset. The stroma tissue segmentation is conducted precisely as can be inferred from the images. For each image, the corresponding ground truth (mask) and the network output is compared in this image. The 98% accuracy over the whole dataset, which is a diverse pile of images from 63 different cancer imaging slides, demonstrates the ability of the proposed U-Net network in segmenting the desired classes and excluding the stroma from the background. This process is conducted according to the IIOBWG guidelines, and thus it can be used for further stroma assessments in research and clinical applications.

### 4.2. TILs Detection

To detect the TILs in stroma tissue, a Mask R-CNN model was employed as per Section 3.2. However, due to the very large number of trainable parameters for the Mask R-CNN model, we used transfer learning. As a starting point for model implementation of the Mask R-CNN, the “mask_rcnn_coco.h5” frozen weights were applied to the model to train it for the first epochs, after training the model for starting epochs, the weights of the Mask R-CNN model were saved and used for the next epochs. The transfer learning process was necessary to get the best results from the Mask R-CNN since its weights have a large size and it was not possible to simulate all the epochs all at once. 

**Figure 7:**
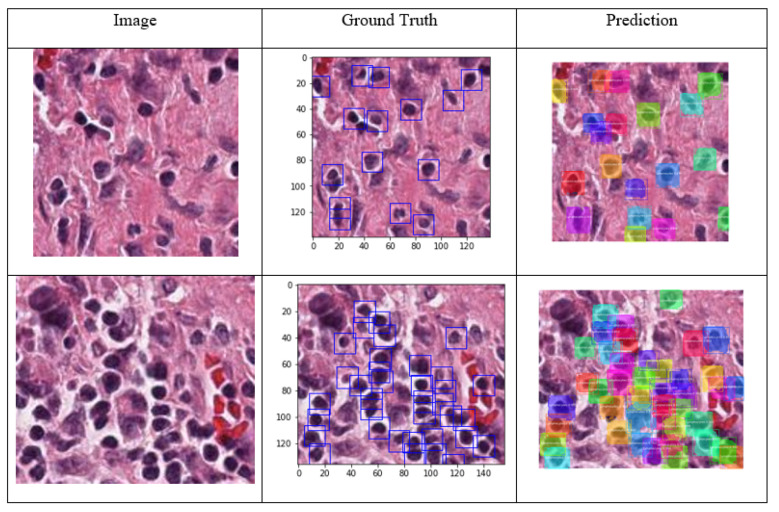
Mask R-CNN results for different sample images with the ground truth (Original magnification: 40×). Different colors indicate different TIL instances distinguished individually.

Since the number of images, and the image input size for the Mask R-CNN can be different, the number of steps per epoch needs to be specified, the number of steps per each epoch was set to 100 for our Mask R-CNN model to allow the network to learn the features efficiently. The Mask R-CNN results for TILs detection are presented in Figure 7. The input image and the target are shown in the figure in the first two columns, and the TILs detected are displayed in the last column. In the results, the class name for each instance is shown on the top of the mask created by Mask R-CNN. Here, there is only one class (lymphocytes), thus, all the segmented objects belong to the lymphocytes. Also, the prediction confidence for all the detected TILs is shown near the name of the objects. The object detection confidence was set to be above 70% for the TILs detection.

Since each TIL cell was labeled separately, this detection can be considered as instance segmentation of the TILs. The segmentation of each object was labeled as one specific class; and even though there could be more than one object in each class, each object is segmented and localized individually. By evaluating the results from Mask R-CNN output, we could see that all the TIL cells are detected correctly (true positive), however, there are some cells segmented as TILs, but in the ground truth, they are not labeled (false positive). This result is acceptable since the tissue structure and cells are very similar. False positive detection is a common flaw in detection algorithms, and it occurs when the objects have similar texture, shape, color, or visual pattern. There are several methods to combat the false positive in object detection, including adding the number of images in the dataset, using more sophisticated algorithms, and tuning the hyperparameters [35,36]. Since the Mask R-CNN is one of the most sophisticated models in object detection, we tried to eliminate false positive detection by expanding the dataset for the TILs from initial 8000 annotations to 12,000 as well as training the Mask R-CNN model for more epochs. To evaluate the performance of TILs detection, more than 1200 TILs in the images were used to quantify the performance of the detection. A total of 1156 cells were correctly labeled as TILs and were considered as true positives (TP). The model failed to segment 17 labeled cells as TILs which were the false negative (FN) predictions. Moreover, 127 objects were identified as TILs incorrectly which were the false positive (FP) predictions. Hence, there was only one class (TILs) to identify; the true negative predictions were not applicable. The final accuracy of the segmentation of the TILs is calculated as follows:

(2) $$Accuracy=\frac{TP}{TP+FP+FN}=\frac{1156}{1156+127+17}=0.889$$

The accuracy of the detection of the TILs is 88.9% and the confusion matrix is shown in Table 2.

### 4.3. Validation and Statistical Analysis

For the final evaluation of the TILs detector software, the TILs scores assigned to each slide by the software were compared with the scores assigned by pathologists to the slides. Two pathologists from the TTUHSC individually assigned an overall score to the slides from the patients. Then, the average score was considered the final TILs score for each scanned slide. The TILs scores from the implemented deep learning model, TILs detector software, were compared with these average scores using statistical analysis methods.

The developed model performance was accurate in cancer slides with high TILs percentage and the difference between the assigned percentages from the pathologists, and the score from the software were small in the slides with a high number of TILs. The difference between the assigned values from the pathologists, and the obtained values from the software showed a higher difference in slides with a very number of TILs (TILs percentage less than 10%). This difference in TILs score for slides with low percentages does not make a clinical difference as these small TILs values are clinically treated the same. The average difference between the results from the software and the pathologists is 54.2%. This large difference in value is due to the fact that for the cancer slides with a low number of TILs, even a small difference causes a large percentage difference. For example, in one slide, the pathologists assigned 1.5%, while the score from the software was 3.0% which means there is a 100% difference. However, this 1.5% difference in TILs scoring does not have a big clinical difference. Thus, the result from the TILs detector software is feasible. Furthermore, when the performance of the developed model is evaluated, these differences are expected, and the most important aspect of evaluation is that the results from the pathologists and the software agree with each other. To elaborate, we expect a small score for the cancer slides with few TILs and a high score for cancer slides with a high number of the TILs which is demonstrated by the statistical analysis in the following section.

**Figure 8:**
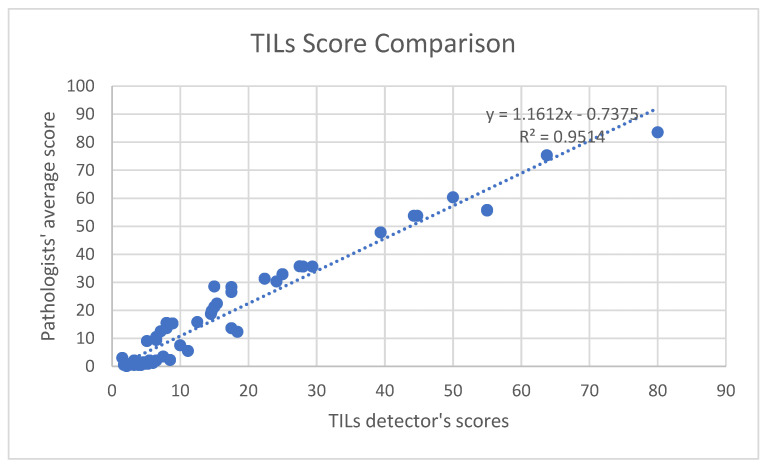
TILs score comparison between pathologists’ average and the proposed pipeline.

Statistical analyses indicated that the TILs scores from the software are in concord with the average scores from the pathologists. The correlation coefficient, R-value, for the comparison was 0.9754 and the R-square was 0.9514, which is shown in Figure 8. This confirms the strong correlation between the TILs scores from the software and the given values from pathologists. Furthermore, the *p*-value of the comparison is 0.045 (<0.05), which shows that the output scores from the software are statistically significant.

## 5. Conclusions

We used computational biology and deep learning models to explore and replicate the scoring of the TILs biomarker in cancer treatment procedures. The development of the two-stage pipeline for detecting and scoring the TILs at a microscopic and cellular level was based on the IIOBWG guidelines. This is the first time that this two-stage approach to score TILs in large, scanned whole breast tumor slides was conducted. The implemented method of scoring TILs in cancer slides with high zoom level and assigning the final TILs score based on the methodology that pathologists use in their research and cancer treatments was another novelty of this research. The statistical analysis confirmed the agreement of the implemented approach of combining two deep learning methods to score TILs in pathology slides with the approach being currently used in clinical and research applications. Moreover, a TILs detection GUI was also developed, since one of the main goals of this manuscript was to generate tools that employ the most accurate deep learning models for biomedical and computational pathology researchers and medical doctors. With the use of the GUI, researchers can load an image to the Mask R-CNN model, detect the TILs and get the TILs scored automatically. Consequently, the suggested deep learning pipeline can perform the TILs scoring task accurately which was the main goal of the paper. We hope that demonstrating the ability of the proposed deep learning method to detect the TILs in breast cancer slides will motivate future research in this field to conceptualize and formulate the TILs scoring procedures in other cancer types based on the IIOBWG or other recognized guidelines.

## 6. Study Limitations and Future Work

Since in this study we collaborated with two pathologists, one of the main limitations of the study was verifying the TIL scores for each slide. Accurately scoring histopathology breast cancer slides was a critical step in the development of the deep learning models and having the slides scored by more pathologists could increase the accuracy of TIL scores. However, because of the limited number of pathologists who worked on the slides in this study, we had to overcome this limitation and establish a procedure to ensure the accuracy of the TIL scores from the pathologists. To do so, each pathologist assessed the slides individually, and the average score from the pathologist was considered the ground truth TILs score for each cancer slide. If the difference between the scores assigned to the slides by pathologists was too high, for example, one scored a low percentage for a slide and the other researcher scored a high percentage, we asked the pathologists to assess the slide together and verify a TILs score for the slide that they both agree on.

Another main limitation of the study was the small number of the cancer slides used, as we had only access to 63 whole cancer slides. The patching method was used to create tiles that were sufficient to detect the stroma and the TILs. This created a sufficient dataset for the training process of the deep learning models at the cellular level. Nevertheless, the number of whole cancer slides was not adequate to develop and train a deep learning model with the final TILs scores from the pathologists. In this regard, statistical analyses were conducted to confirm the concordance of the results from the software with the assessment from the pathologists. 

For future applications, we would acquire additional whole cancer slides from other pathologists or cancer centers along with their scores. Those scores could be used to further consolidate the ground truth we obtained in our study. Furthermore, the developed models could be further trained, analyzed, and validated with more cancer slides to make the model more inclusive, rigorous, and robust, to allow its usage in future clinical applications.

## Figures and Tables

**Table 1:** Time comparison for GUP and CPU.

Processor Type	Time(s)
CPU	55.35
GPU	2.79

**Table 2:** The confusion matrix of the Mask R-CNN. The green color shows the correct predictions while the red shows the incorrect detections.

	Predicted TILs	Predicted NOT TILs
Actual TILs	1156	17
Actual NOT TILs	127	N/A

## Data Availability

Any data request should be directed to the corresponding author, Dr. Hanna Moussa (hanna.moussa@ttu.edu).
